# First Experience in the Control of the Venous Side of the Brain AVM

**DOI:** 10.3390/jcm10245771

**Published:** 2021-12-09

**Authors:** Stephan Waldeck, Rene Chapot, Christian von Falck, Matthias F. Froelich, Marc Brockmann, Daniel Overhoff

**Affiliations:** 1 Department of Diagnostic and Interventional Radiology and Neuroradiology, Bundeswehr Central Hospital Koblenz, Rübenacher Straße 170, 56072 Koblenz, Germany; daniel.overhoff@umm.de; 2 Institute of Neuroradiology, University Medical Centre, Johannes Gutenberg University Mainz, Langenbeckstraße 1, 55131 Mainz, Germany; mbrockma@uni-mainz.de; 3 Department of Neuroradiology, Alfried Krupp Krankenhaus, Alfried-Krupp-Strasse 21, 45131 Essen, Germany; rene.chapot@krupp-krankenhaus.de; 4 Institute of Diagnostic and Interventional Radiology, Hannover Medical School, Carl-Neuberg-Straße 1, 30625 Hanover, Germany; vonfalck@icloud.com; 5 Department of Radiology and Nuclear Medicine, University Medical Centre Mannheim, Medical Faculty Mannheim, Heidelberg University, Theodor-Kutzer-Ufer 1-3, 68167 Mannheim, Germany; matthias.froelich@umm.de

**Keywords:** cerebral arteriovenous malformation, pressure cooker technique, transvenous approach

## Abstract

Background and purpose: Brain arteriovenous malformations (AVM) are increasingly curable with endovascular embolization. This study examines the preliminary experience with a novel double-sided hybrid approach in the treatment of cerebral arteriovenous malformations (AVM) versus a purely single-sided intra-arterial approach. Materials and methods: The single-center study cohort included 18 patients with brain AVMs (Spetzler–Martin Grade 2 or 3) having stand-alone endovascular treatment with either the arterial-side-only pressure cooker technique (aPCT) (group 1; *n* = 9) or a double-sided hybrid intra-arterial and transvenous approach (HIPRENE) (group 2; *n* = 9). Results: Patients belonging to group 2 had lower rates of intra-procedural hemorrhaging (66.7% vs. 33.3%, *p* = 0.169) and needed fewer treatment sessions to achieve nidus occlusion (1.7 vs. 1.2, *p* = 0.136). The HIPRENE treatment regime led to higher nidus occlusion rates after the initial treatment compared to aPCT (77.7% vs. 44.4%, *p* = 0.167). Group 2 patients had a lower rate of neuromonitoring events (22.2% vs. 44.4%, *p* = 0.310) and fewer accounts of blood flow obstruction in post-operative MRIs (33.3% vs. 55.6%, *p* = 0.319). Conclusion: A double-sided hybrid intra-arterial and transvenous approach might have benefits for curative endovascular brain AVM treatment in patients with Spetzler–Martin Grade 2 or 3. In our small study cohort, the HIPRENE treatment regime had higher nidus occlusion rates after the first treatment, which reduces the number of treatment sessions and lowers intra- and post-operative complication rates. Further randomized controlled studies are awaited to corroborate our preliminary outcomes.

## 1. Introduction

Cerebral arteriovenous malformations (AVMs) are curable with endovascular embolization [1]. Traditionally, endovascular neuroradiologists favor an intra-arterial approach using liquid embolic agents such as cyanoacrylates or copolymers. Out of these, the precipitating hydrophobic injectable liquid has become increasingly popular due to their extended polymerization times. This characteristic enables longer injection times, thus providing a more controlled embolization and improved penetration of the target malformation [2].

Copolymer injection is largely performed using the so-called plug-and-push technique, in which the copolymer forms a plug around the tip of the microcatheter, thereby facilitating forward penetration of the embolization agent [3]. However, the degree of reflux is difficult to control and may lead to occlusion of non-target arteries with potentially fatal outcomes due to ischemic stroke or hemorrhaging [4,5]. New trends in embolization techniques, such as transarterial balloon-assisted embolization, transvenous embolization, and the transarterial pressure cooker technique (PCT), effectively reduce copolymer reflux [3].

The PCT relies on a second microcatheter to create an anti-reflux plug consisting of acrylic glue and injectable or detachable coils around the detachable portion of the primary - ethylene vinyl alcohol (EVOH) copolymer microcatheter. The anti-reflux plug allows for wedge-flow conditions and a more forceful EVOH copolymer injection while hindering EVOH copolymer reflux [6]. However, premature venous occlusion with nidus engorgement and hemorrhaging remains a major challenge of the PCT [7].

Transvenous embolization offers unique advantages over the trans-arterial approach in cases with extremely distal nidus locations and/or complex arterial anatomy [8]. However, problems may arise in cases with patent arterial feeders as the risk of rupture is increased.

The endovascular AVM combination treatment regimes described in the literature refer to endovascular treatment in conjunction with either radiosurgery or with microsurgical resection. However, a double endovascular treatment approach allowing intraoperative control of both the afferent and efferent vessels of the nidus has yet to be described.

The study’s aims were twofold, namely:

(i) To present the novel combination of simultaneous intra-arterial and transvenous approaches, named the hybrid high-pressure nidus embolization (HIPRENE);
(ii) To analyze brain AVM treatment outcomes using an intra-arterial-only approach with high PCT compared to the HIPRENE treatment regime with a double-sided hybrid approach.

## 2. Materials and Methods

The local ethics committee approved this retrospective cohort preliminary study.

### 2.1. Patients

**Figure 1:**
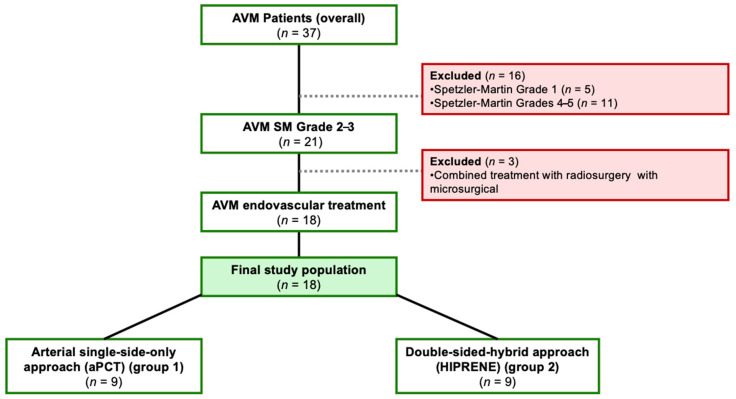
Flowchart of the inclusion and exclusion criteria. AVM arteriovenous malformation; SM Spetzler Martin; aPCT arterial pressure cooker technique/single-sided arterial approach; HIPRENE hybrid high-pressure nidus embolization/double-sided hybrid approach.

Primarily, 37 patients were included in this single-center study cohort, of which 26 patients with low- and intermediate-grade brain AVMs were treated with endovascular embolization between October 2014 and August 2021 in a maximum care hospital. High-grade (Spetzler–Martin (SM) grades 4–5) AVMs were not taken into consideration because of the high complexity of their treatment, their volume, complex angioarchitecture, and often critical localization. We excluded eight patients with low-grade AVM (Grade 1) on the Spetzler–Martin Grading Scale and those having combined endovascular embolization with radiosurgery or microsurgical resection. The exclusion criteria can be seen in Figure 1.

### 2.2. Methods

A total of 18 patients (10 females and 8 males) with either intra-arterial or transvenous embolization brain AVMs were included in the analyses. The mean age of the females was 54.5 ± 19.4 years (median: 55 years), and that of males was 47.5 ± 15.8 years (median: 45.5 years). We extracted the following data from these patients’ records: patient identifier, date of birth, sex, Spetzler–Martin grade, nidus size, incidence of hemorrhaging, number of treatments, number of venous drains, number of arterial feeders, liquid embolization agent, pressure cooker agent, post-operative nidus occlusion rate, incidence of post-operative complications, and residual brain AVM. Nine patients were treated with the single-sided arterial approach (aPCT), while the other nine patients were treated with the double-sided hybrid approach (HIPRENE) regime.

### 2.3. Embolization Procedure

A team of experienced interventional neuroradiologists performed all interventions under general anesthesia. During embolization, blood pressure in both groups was lowered by the anesthesiologist to a systolic value below 80 mmHg with medication. No special company-related proctoring for the procedures was required.

#### 2.3.1. Arterial Single-Side-Only Approach (aPCT) (Group 1)

A dimethyl sulphoxide-compatible microcatheter (detachable tip: Apollo, Ev3, Irvine, California, USA or Sonic, Balt Extrusion, Montmorency, France//non-detachable tip: Echelon-10, Ev3-Covidien, Dublin, Ireland) was advanced to the respective feeder artery. A second microcatheter (Magic 1.2FM, Balt Extrusion, Montmorency, France or Echelon-10) was positioned alongside the primary microcatheter, with its tip slightly further distal to the detachment zone marking. The plug was formed by injecting coils and a subsequent mixture of acrylate glue (Histoacryl, B. Braun, Melsungen, Germany) and iodized oil (Lipiodol Ultra Fluide, Guerbet, France) in a ratio of 1:2 through the secondary microcatheter. The second microcatheter was removed after coil/glue delivery and plug completion. Following plug formation, continuous anterograde injection of the Onyx 18 (Medtronic, Irvine, California, USA) was performed via the primary microcatheter. Whenever possible, the primary microcatheter was withdrawn completely after nidus occlusion. In cases of microcatheter entrapment, however, the detachable tip remained in situ. Following the intervention, patients underwent post-operative MRI and neurological monitoring while hospitalized.

#### 2.3.2. Double-Sided Hybrid Approach (HIPRENE) (Group 2)

The double-sided hybrid HIPRENE treatment regime consisted of a primary venous PCT plug. Transvenous retrograde nidus sclerotherapy under controlled hypotension (TRENSH) relies on either temporary systemic hypotension or occlusion of the main arterial feeders. The induced hypotension leads to nidus decompression and reverses the intranidal pressure gradient, thereby allowing for retrograde filling of the nidus [9].

**Figure 2:**
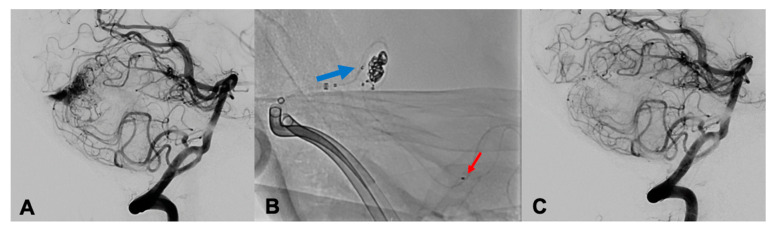
Double-sided access for the HIPRENE procedure; (**A**) Initial findings, lateral view of the AVM. (**B**) The blue arrow points to the nidus embolization via the venous side; the red arrow points to the arterial microcatheter for the double-sided hybrid approach. (**C**) Final control lateral view of the AVM.

Trans-arterial microcatheters for the double-sided approach were positioned and primed for arterial embolisation, but only used if necessary. Two guiding catheters were initially placed in the cervical internal artery and intracranial venous sinus. Both the primary and secondary microcatheters were carefully advanced to the draining vein of the nidus, and endovascular plug formation ensued. The plug was formed with coils, and Histoacryl or Magic Glue (Balt Extrusion, Montmorency, France) and Onyx 18 or Squid 12 (Balt Extrusion, Montmorency, France) were used as liquid embolic agents. Figure 2 shows the double-sided access for the HIPRENE procedure.

The injection was commenced via the transarterial primary microcatheter to reduce nidus inflow and decrease the pressure gradient, before following with the retrograde injection aimed at nidus obliteration. After nidus occlusion, all microcatheters were removed, and post-operative procedures were performed as stated above for group 1.

### 2.4. Statistical Analysis

The extracted patient data was compiled in an Excel spreadsheet. SPSS (IBM SPSS Statistics, version 20.0 for Macintosh; SPSS, Inc., Chicago, IL, USA) was used for statistical calculations. Independent variables were analyzed with the Mann–Whitney U test, and the Chi-squared test was used in comparisons involving categorical variables. A *p*-value of *p* < 0.05 was considered significant. No sample sizing was considered, as the study was retrospective and no randomization was possible.

## 3. Results

### 3.1. Baseline Characteristics

In group 1, nine patients (55.6% male, 44.4% female) with a mean age of 50.75 ± 18.75 years (median: 54 years), had endovascular embolization with a single-sided transarterial approach (aPCT). Six of these patients (66.5%) presented with Spetzler–Martin grade II, while the three remaining patients (33.5%) had grade III AVMs. Nidus diameters ranged from 11 to 41 mm, with an average size of 27.2 ± 8.7 mm (median: 26 mm).

In group 2, the nine patients (33.3% male, 66.7% female) with a mean age of 48.33 ± 16.02 years (median: 45 years) were treated with a double-sided hybrid approach (HIPRENE). Five patients (55.6%) had Spetzler–Martin grade II and the other four (44.4%) patients presented with grade III AVMs. The mean nidus diameter was 23.2 ± 9.2 mm (median: 25 mm) and ranged from 11 to 38 mm. An overview of the baseline characteristics is given in Table 1.

### 3.2. Arterial Single-Side-Only Approach (aPCT) (Group 1)

Two-thirds (66.5%) of the patients presented with two feeder arteries, and the remaining third (33.5%) had only one feeder artery. The majority (*n* = 5; 55.6%) of those treated had two draining veins. Three draining veins were the second most common configuration (*n* = 3, 22.2%), and one patient (11.2%) had only one draining vein. The liquid embolic agent Onyx 18 was used for nidus occlusion in all nine patients belonging to group 1. The endovascular plug consisted of coils and glue in eight (88.8%) cases and one patient (11.2%) had a plug without glue. Cerebral hemorrhaging before the intervention occurred in six patients (66.7%), whereas three aPCT patients (33.3%) did not experience hemorrhaging before the treatment.

A single aPCT treatment session was sufficient to achieve nidus obliteration in four patients (44.4%), while another four patients (44.4%) required retreatment. One patient (11.2%) needed three interventions to reach nidus occlusion. Neuromonitoring led to the detection of events in four patients (44.4%). Post-operative MRIs revealed the presence of diffusion-weighted lesions in five (55.6%) of the patients treated.

### 3.3. Double-Sided Hybrid Approach (HIPRENE) (Group 2)

Five patients (55.6%) in the HIPRENE group had two feeder arteries, and four patients (44.4%) had only one feeder artery. Two draining veins were the most common venous configuration in five patients (55.6%), followed by one and three draining veins in two patients each (22.2%). The primary embolization agent Onyx 18 was used in the majority (*n* = 7; 77.7%) of patients in group 2, and only two (22.3%) had Squid 12 injections. Coils and Histoacryl glue were used for plug formation in eight (88.8%) patients, and one patient (11.2%) had coils and Magic Glue. Three patients (33.3%) experienced cerebral hemorrhaging before the intervention.

Under the double-sided hybrid approach, complete nidus occlusion was achieved after only one treatment in seven (77.7%) patients. The remaining two (22.3%) patients underwent a second session before the nidus was successfully obliterated. Neuromonitoring led to the detection of events in two patients (22.2%). Diffusion-weighted lesions were detected in three patients (33.3%) following post-operative MRI.

### 3.4. Comparison of Treatment Outcomes

The differences between the two approaches were non-significant with regard to pre-procedural hemorrhage (*p* = 0.169) and the number of treatment sessions until nidus occlusion (*p* = 0.136). Comparison of treatment outcomes for nidus occlusion rates following the initial treatment session, neuromonitoring event rates, and amount of diffusion-weighted lesions in post-operative MRIs revealed no statistically significant differences (Table 2).

## 4. Discussion

This preliminary retrospective cohort study aimed to compare treatment outcomes for traditional arterial-side-only versus a novel double-sided hybrid (HIPRENE) approach in curative brain AVM treatment. Although a learning curve was necessary due to the complexity of the double-sided catheterization, the HIPRENE treatment regime led to superior results after only one treatment session and lowered the rate of both intra- and post-operative complications. The fact that the comparison of treatment outcomes did not reach statistical significance is most likely attributable to the small sample size in this study.

In general, transvenous nidus occlusion is becoming increasingly more popular in AVM treatment and represents a safe and curative option for selected AVM, with nidus occlusion rates ranging from 56 to 100% in the literature [10,11]. While a review of transvenous AVM techniques cautiously described the primary indication as a salvage therapy in patients with otherwise untreatable lesions or deep AVMs due to the limited number of articles available [8], more recent studies suggest an extension of indications to include curative treatment of superficial AVMs [12,13]. Recent reports also show promising evidence for transvenous embolization as a viable curative treatment option in patients with high-grade AVMs [14,15]. In contrast, the results of this study show that 100% curative treatment of grade 2–3 AVMs is achievable via transvenous nidus occlusion in an average of 1.2 treatment sessions. In view of these outcomes, the indications for primary curative AVM treatment via transvenous embolization could be potentially extended further in the future.

The unique combination of simultaneous intra-arterial and transvenous catheterization presented in this study allows for more intra-operative flexibility in that the treatment regime may be switched or combined spontaneously, if necessary. This option is especially valuable in complex cases. However, seemingly straightforward cases may also benefit from the double-sided hybrid approach, since even the most careful preoperative mapping and treatment planning does not allow complete mapping of the nidus, all feeding arteries, and venous drains [16].

Currently, only one study reports on the use of double transarterial and transvenous embolization. The report describes using the intra-arterial microcatheter to inject a liquid embolic agent to reduce nidus inflow before a subsequent transvenous injection for retrograde filling [17]. Unfortunately, the study does not include information on the number of patients treated with this method or on individual outcomes.

## 5. Study Limitations

This study is limited mainly by its small sample size of patients from a single center and by the retrospective analysis of the available data. Our cohort, non-randomized study focused on a niche of the whole neuroradiology practice with a relatively low number of patients. This may represent an issue in the statistical validity of the comparison of the two groups. Outcome analysis with respect to clinical parameters such as length of hospitalization and late post-operative complications for the different study cohorts was not the primary target of this study. Future randomized studies, with larger patient cohorts from multiple centers, are warranted in order to confirm our findings and to be able to extend the indications for curative AVM treatment via transvenous embolization.

## 6. Conclusions

Our preliminary experience with the novel double-sided hybrid intra-arterial and transvenous approach (HIPRENE) suggests that several advantages may emerge over the traditional single-sided arterial treatment regimen in the curative endovascular treatment of Spetzler–Martin Grade 2 or 3 brain AVMs.

Compatibly with the limitations of our study, our clinical experience confirms that the HIPRENE treatment regimen had higher post-treatment nidus occlusion rates after the first session in this small patient cohort. There was no significant difference in the number of neurological events between the two treatments. We do not know if this is due to the small number of treatment cases, and more studies are needed to confirm these preliminary results.

In addition, the double-sided hybrid approach allowed for a dynamic switch of arterial or venous approaches during the procedure, which could be particularly beneficial in more complex cases.

## Figures and Tables

**Table 1:** Baseline characteristics of treatment groups.

	Group 1 (*n* = 9)	Group 2 (*n* = 9)
Males (*n*/%)	5/55.6	3/33.3
Age/years (median)	54	45
Spetzler–Martin grade II (*n*/%)	6/66.5	5/55.6
Spetzler–Martin grade III (*n*/%)	3/33.5	4/44.4
Nidus diameter/mm (median)	26	25

**Table 2:** Comparison of treatment outcomes.

	Group 1	Group 2	*p*-Value
Pre-procedural hemorrhaging (%)	66.7%	33.3%	*p* = 0.169
Number of treatment sessions to nidus occlusion (Ø)	1.7	1.2	*p* = 0.136
Nidus occlusion rate after initial treatment session (%)	44.4%	77.7%	*p* = 0.167
Neuromonitoring event rate (%)	44.4%	22.2%	*p* = 0.310
Diffusion weighted lesions in post-operative MRI (%)	55.6%	33.3%	*p* = 0.319

MRI—magnetic resonance imaging.

## Data Availability

The data presented in this study are available in the article.

## Abbreviations

AVM	Arteriovenous malformation
HIPRENE	Hybrid high-pressure nidus embolization
NBCA	*N*-butyl-2-cyanoacrylate
PCT	Pressure cooker technique
TRENSH	Transvenous retrograde nidus sclerotherapy under controlled hypotension
